# Polymorphisms in the *TMEM132D* region are associated with panic disorder in HLA-DRB1*13:02-negative individuals of a Japanese population

**DOI:** 10.1038/hgv.2016.1

**Published:** 2016-02-25

**Authors:** Mihoko Shimada-Sugimoto, Takeshi Otowa, Taku Miyagawa, Seik-Soon Khor, Yosuke Omae, Licht Toyo-oka, Nagisa Sugaya, Yoshiya Kawamura, Tadashi Umekage, Akinori Miyashita, Ryozo Kuwano, Hisanobu Kaiya, Kiyoto Kasai, Hisashi Tanii, Yuji Okazaki, Katsushi Tokunaga, Tsukasa Sasaki

**Affiliations:** 1 Department of Human Genetics, Graduate School of Medicine, The University of Tokyo, Tokyo, Japan; 2 Graduate School of Clinical Psychology, Teikyo Heisei University Major of Professional Clinical Psychology, Tokyo, Japan; 3 Department of Psychiatry and Behavioral Sciences, Tokyo Metropolitan Institute of Medical Science, Tokyo, Japan; 4 Department of Epidemiology and Public Health, Graduate School of Medicine, Yokohama City University, Kanagawa, Japan; 5 Department of Psychiatry, Shonan Kamakura General Hospital, Kanagawa, Japan; 6 Division for Environment, Health and Safety, The University of Tokyo, Tokyo, Japan; 7 Department of Molecular Genetics, Center for Bioresources, Brain Research Institute, Niigata University, Niigata, Japan; 8 Panic Disorder Research Center, Warakukai Med. Corp., Tokyo, Japan; 9 Department of Neuropsychiatry, Graduate School of Medicine, The University of Tokyo, Tokyo, Japan; 10 Department of Psychiatry, Institute of Medical Life Science, Graduate School of Medicine, Mie University, Mie, Japan; 11 Tokyo Metropolitan Matsuzawa Hospital, Tokyo, Japan; 12 Department of Physical and Health Education, Graduate School of Education, The University of Tokyo, Tokyo, Japan

## Abstract

We herein report an association between *TMEM132D* and panic disorder (PD) in a Japanese population, evaluating the effects of HLA-DRB1*13:02, which we previously reported as a susceptibility genetic factor for PD. SNPs in *TMEM132D* showed significant associations with PD in subjects without HLA-DRB1*13:02 (rs4759997; *P*=5.02×10^−6^, odds ratio=1.50) but not in those with the *HLA* allele. *TMEM132D* might have a role in the development of PD in subjects without HLA-DRB1*13:02.

Panic disorder (PD) is an anxiety disorder characterized by panic attacks and anticipatory anxiety. PD is relatively common; the lifetime prevalence is reported to be 1–3%.^1^ According to a previous twin study, the heritability of PD is estimated to be 0.43,^2^ which suggests that both genetic and environmental factors have a role in the pathogenesis of PD. To date, several studies that applied a candidate-gene approach have reported susceptibility genes of PD, but many of them have not been successfully replicated in subsequent studies.^3^ Recently, a genome-wide association study (GWAS) of European ancestry identified single-nucleotide polymorphisms (SNPs) in the transmembrane protein 132D gene (*TMEM132D*) associated with PD.^4^ This result was supported by a replication study and meta-analyses of European subjects, which confirmed that *TMEM132D* is a susceptibility gene of PD.^5,6^ However, in a Japanese GWAS of PD, SNPs in *TMEM132D* did not show a positive association with PD.^7,8^

We previously found associations between PD and human leukocyte antigen (HLA), especially the *HLA-B* and *HLA-DRB1* genes, based on pathway analyses using the results from our Japanese GWAS of PD.^8^ HLA is the human version of the major histocompatibility complex, which presents endogenous antigens to CD8+ and CD4+ T cells. There is a great number of polymorphisms in the *HLA* genes. *HLA* genes have been reported to be involved in not only immune-related diseases^9^ but also several psychiatric disorders.^10^ We genotyped the *HLA-B* and *HLA-DRB1* genes, and confirmed that the frequency of HLA-DRB1*13:02 was significantly higher in PD patients than in healthy individuals (case positivity: 18.1%; control positivity: 11.5%; *P*=2.62×10^−5^; odds ratio (OR)=1.70).^11^

Previous studies have reported that the genetic factors and clinical features of several *HLA*-associated diseases differ between *HLA* allele-positive and -negative patients. Narcolepsy, with and without cataplexy, was associated with HLA-DQB1*06:02,^12^ and the severity of narcolepsy without cataplexy was higher in HLA-DQB1*06:02-positive patients than in HLA-DQB1*06:02-negative patients.^12,13^ HLA-B*51 was strongly associated with risk factors for Behçet’s disease,^14^ and a significant association between one SNP in the *ERAP1* locus was observed only in HLA-B*51-positive patients.^14^ Hence there is a possibility that the genetic backgrounds might differ in PD subjects with or without HLA-DRB1*13:02. To account for these effects of *HLA* alleles, we focused on a candidate PD gene, *TMEM132D*, and investigated the SNPs in the *TMEM132D* region in both HLA-DRB1*13:02-positive and -negative subjects. In this analysis, genotyping data for the SNPs were generated using the Genome-Wide Human SNP Array 6.0 (Affymetrix, Santa Clara, CA, USA). Inclusion criteria for quality control were SNP call rate >0.95, Hardy–Weinberg equilibrium (HWE) test *P*>0.001, and minor allele frequency (MAF)>0.05. We defined ‘gene region’ as the region located 50 kb upstream to 50 kb downstream of *TMEM132D* (chr12: 129556271–130388212 (GRCh37/hg19)). The SNP genotype data were subdivided into two data sets, those of HLA-DRB1*13:02-positive subjects (cases: *N*=103; controls: *N*=198) and those of HLA-DRB1*13:02-negative subjects (cases: *N*=438; controls: *N*=1,341). An imputation analysis was also performed to evaluate the potential association of ungenotyped SNPs in the *TMEM132D* region of both subgroups. IMPUTE2 software^15^ was used to estimate SNP genotypes using the reference data set from 1000 Genomes Phase 3 haplotypes.^15^ We filtered out low-quality imputed SNPs by applying the following conditions: SNP call rate ⩾0.95, HWE test *P*>0.0001, and probability of imputation certainty ⩾0.9. After filtering, a total of 8,070 SNPs remained for subsequent analysis. Using the genotype data of these SNPs, case–control association tests were performed to examine whether SNPs in *TMEM132D* showed an association with PD in each subgroup. We set the significance level after multiple testing correction to *α*=1.26×10^−5^, which was calculated from 0.05 divided by the number of SNPs (*N*=3,978) pruned by high linkage disequilibrium (LD; *r* ^2^>0.8) with PLINK SNP pruning procedure (window size in SNPs=100, the number of SNPs to shift the window=1).^16^

In the analysis of the HLA-DRB1*13:02-negative subgroup, nine SNPs in the *TMEM132D* region showed significant associations, and SNP rs4759997 had the lowest *P* value (*P*=5.02×10^−6^, OR=1.50; Table 1 and Figure 1). In contrast, these SNPs were found to have no association with PD in the HLA-DRB1*13:02-positive group (Table 1 and Supplementary Figure 1). To find other SNPs potentially associated with PD in the HLA-DRB1*13:02-negative group, logistic regression analysis adjusting for the effect of rs4759997 was also performed. The analysis showed that none of the SNPs in the *TMEM132D* region had an association that reached the threshold level of significance, which suggested that the nominal associations of SNPs in this region were derived from LD with rs4759997 (Supplementary Figure 2).

A previous study identified two SNPs, rs7309727 and rs11060369, in *TMEM132D* as susceptibility variants for PD in populations of European ancestry.^4^ The two SNPs were also associated with higher anxiety and larger amygdala volumes.^17^ In addition, the risk genotype of rs11060369 was found to enhance *TMEM132D* mRNA expression in the brain.^4^ These two SNPs identified in populations of European ancestry were located in intron 3 of *TMEM132D*, while the SNPs found in our study, rs4759997 and the surrounding SNPs with significant *P* values, were located in intron 1. The SNP with the lowest *P* value, rs4759997, was not in LD with either rs7309727 or rs11060369 in individuals of Japanese ancestry (Japanese; rs7309727, *r* ^2^=0.001; rs11060369, *r* ^2^=0.003), while in individuals of European ancestry, SNP rs4759997 had very low frequency (MAF=0.009) according to HapMap data.^18,19^ In addition, imputation analysis revealed that the two SNPs, rs7309727 and rs11060369, were not associated with PD in HLA-DRB1*13:02-negative Japanese subjects (rs7309727: case MAF=0.36, control MAF=0.39, *P*=0.124; rs11060369: case MAF=0.46, control MAF=0.46, *P*=0.826). Such results, showing that different SNPs in *TMEM132D* are associated with PD in individual populations, might be derived from differences in the LD structure between the populations of Japanese and European ancestry (Supplementary Figure 3). Therefore, targeted resequencing of this gene is required in a future study.

Our study provides initial evidence that SNPs in *TMEM132D* show significant associations with PD in a HLA-DRB1*13:02-negative group of Japanese individuals. Specifically, *TMEM132D* might affect PD in HLA-DRB1*13:02-negative individuals. Further replication studies in independent and larger *HLA*-typed population samples are required to confirm these associations.

## Figures and Tables

**Figure 1:**
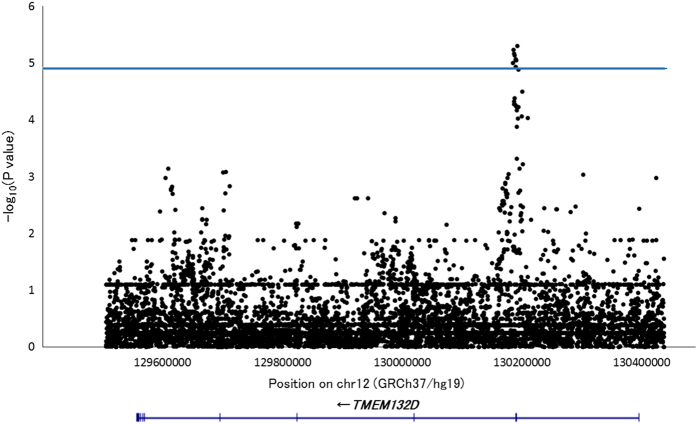
Results of the HLA-DRB1*13:02-negative subgroup analysis in the *TMEM132D* region. Physical positions are based on GRCh37/hg19. The blue line represents the significance threshold (*α*=1.26×10^−5^).

**Table 1:** SNPs with *P*-value <10^−4^ in the *TMEM132D* region

*Position*^a^	*SNP*	HLA-DRB1*13:02 negative				HLA-DRB1*13:02 positive			
		*MAF*		P-*value*	*OR*	*MAF*		P-*value*	*OR*
		*PD*	*Control*			*PD*	*Control*		
130185851	rs1567509	0.283	0.210	1.01×10^−5^ b	1.49	0.211	0.203	0.820	1.05
130186374	rs7311162	0.279	0.205	5.87×10^−6^ b	1.50	0.199	0.198	0.975	1.01
130187014	rs264463	0.105	0.064	4.79×10^−5^	1.73	0.050	0.054	0.854	0.93
130187283	rs1397504	0.281	0.208	6.92×10^−6^ b	1.49	0.199	0.200	0.989	1.00
130187566	rs264464	0.104	0.063	5.30×10^−5^	1.73	0.050	0.054	0.854	0.93
130188352	rs264465	0.105	0.063	4.19×10^−5^	1.73	0.058	0.061	0.908	0.96
130188504	rs7962650	0.279	0.206	7.32×10^−6^ b	1.49	0.194	0.200	0.876	0.97
130189452	rs67208922	0.104	0.063	5.46×10^−5^	1.72	0.050	0.054	0.833	0.92
130189478	rs264468	0.104	0.063	5.46×10^−5^	1.72	0.050	0.054	0.833	0.92
130189868	rs10773696	0.279	0.206	8.65×10^−6^ b	1.49	0.194	0.200	0.876	0.97
130190130	rs7312812	0.279	0.207	1.19×10^−5^ b	1.48	0.194	0.199	0.888	0.97
130190285	rs1510820	0.279	0.207	9.10×10^−6^ b	1.48	0.194	0.200	0.876	0.97
130191111	rs7132791	0.279	0.207	9.10×10^−6^ b	1.48	0.194	0.200	0.876	0.97
130191332	rs264472	0.104	0.063	5.90×10^−5^	1.72	0.050	0.056	0.745	0.88
130191567	rs2398467	0.104	0.063	5.90×10^−5^	1.72	0.049	0.056	0.725	0.87
130191851	rs529395389	0.104	0.063	6.92×10^−5^	1.71	0.049	0.056	0.716	0.87
130192489	rs588761	0.104	0.063	5.90×10^−5^	1.72	0.049	0.056	0.716	0.87
130193038	rs4759997	0.282	0.208	5.02×10^−6^ b	1.50	0.199	0.200	0.989	1.00
130193940	rs663071	0.104	0.064	9.67×10^−5^	1.69	0.049	0.056	0.716	0.87
130195133	rs67408383	0.104	0.063	6.03×10^−5^	1.72	0.049	0.056	0.716	0.87
130195225	rs7304093	0.279	0.208	1.31×10^−5^	1.47	0.194	0.200	0.876	0.97
130199905	rs6486497	0.356	0.286	8.73×10^−5^	1.38	0.257	0.293	0.356	0.84
130201128	rs10744430	0.366	0.292	3.19×10^−5^	1.41	0.277	0.296	0.630	0.91
130210550	rs76801035	0.055	0.027	9.36×10^−5^	2.07	0.025	0.020	0.738	1.21

Abbreviations: MAF, minor allele frequency; OR, odds ratio; PD, panic disorder; SNP, single-nucleotide polymorphism.

a Physical position (according to GRCh37/hg19).

b The significance level after multiple testing correction was set as *α*=1.26×10^−5^.
