# Supplementary material for: Polymorphisms in the TMEM132D region are associated with panic disorder in HLA-DRB1*13:02-negative individuals of a Japanese population
Source: Hum Genome Var. 2016 Feb 25;3:16001–. doi: 10.1038/hgv.2016.1 (PMC4766370; doi:10.1038/hgv.2016.1)
Supplement: Supplemental material [file hgv20161-s1.doc]

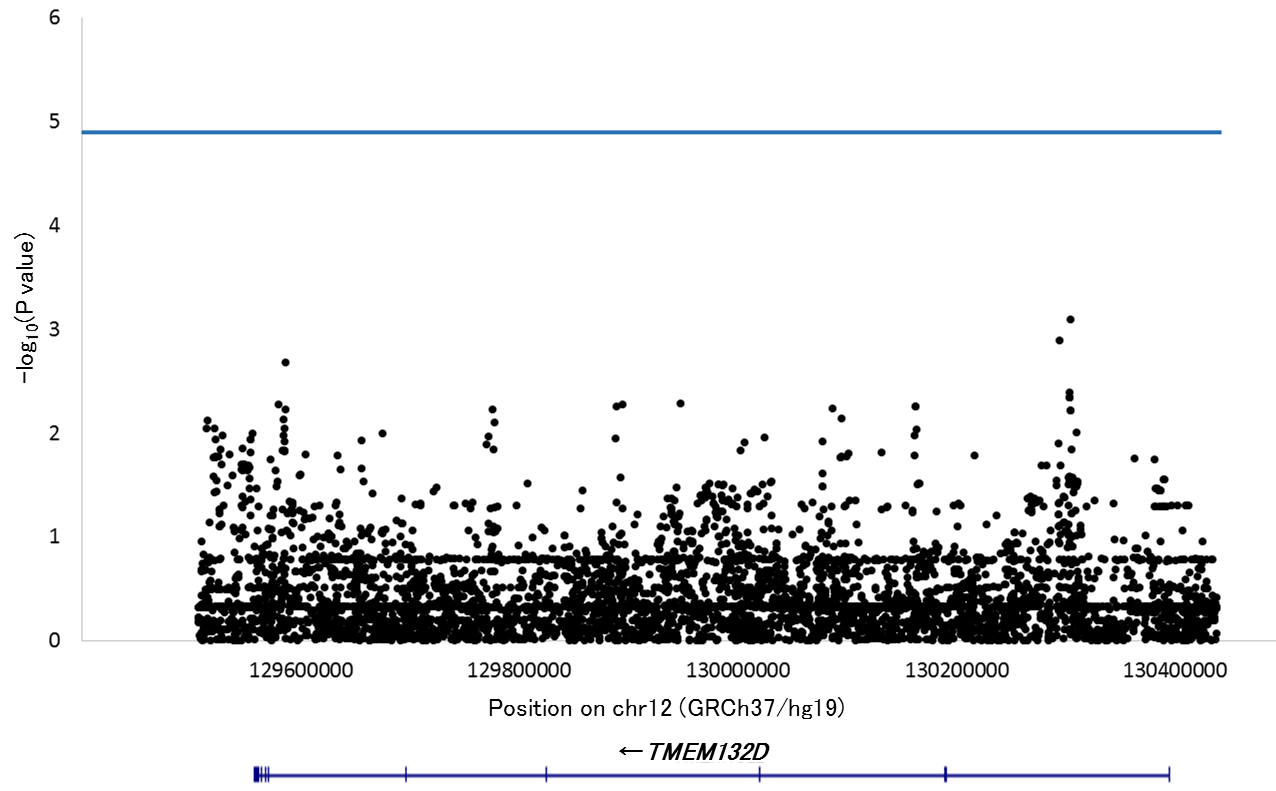
**Supplementary materials**

Supplementary Figure 1. Results of the *HLA-DRB1*13:02*-positive subgroup analysis in the *TMEM132D* region. Physical positions are based on GRCh37/ hg19. The blue line represents the significance threshold (α = 1.26 × 10^-5^).


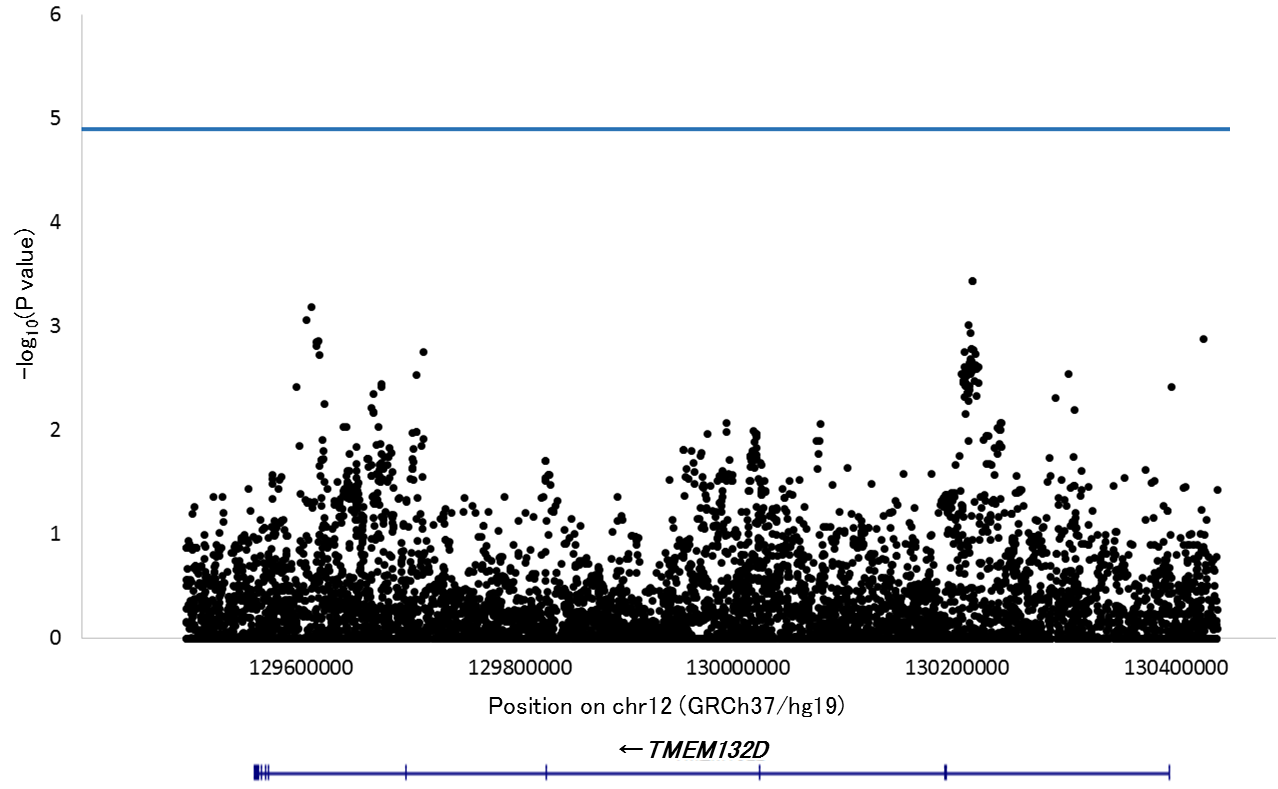


Supplementary Figure 2. Results of the logistic regression analysis of SNPs in the *TMEM132D* region adjusting SNP rs4759997 effect in *HLA-DRB1*13:02*-negative group.

Physical positions are based on GRCh37/hg19. The blue line represents the significance threshold (α = 1.26 × 10^-5^).


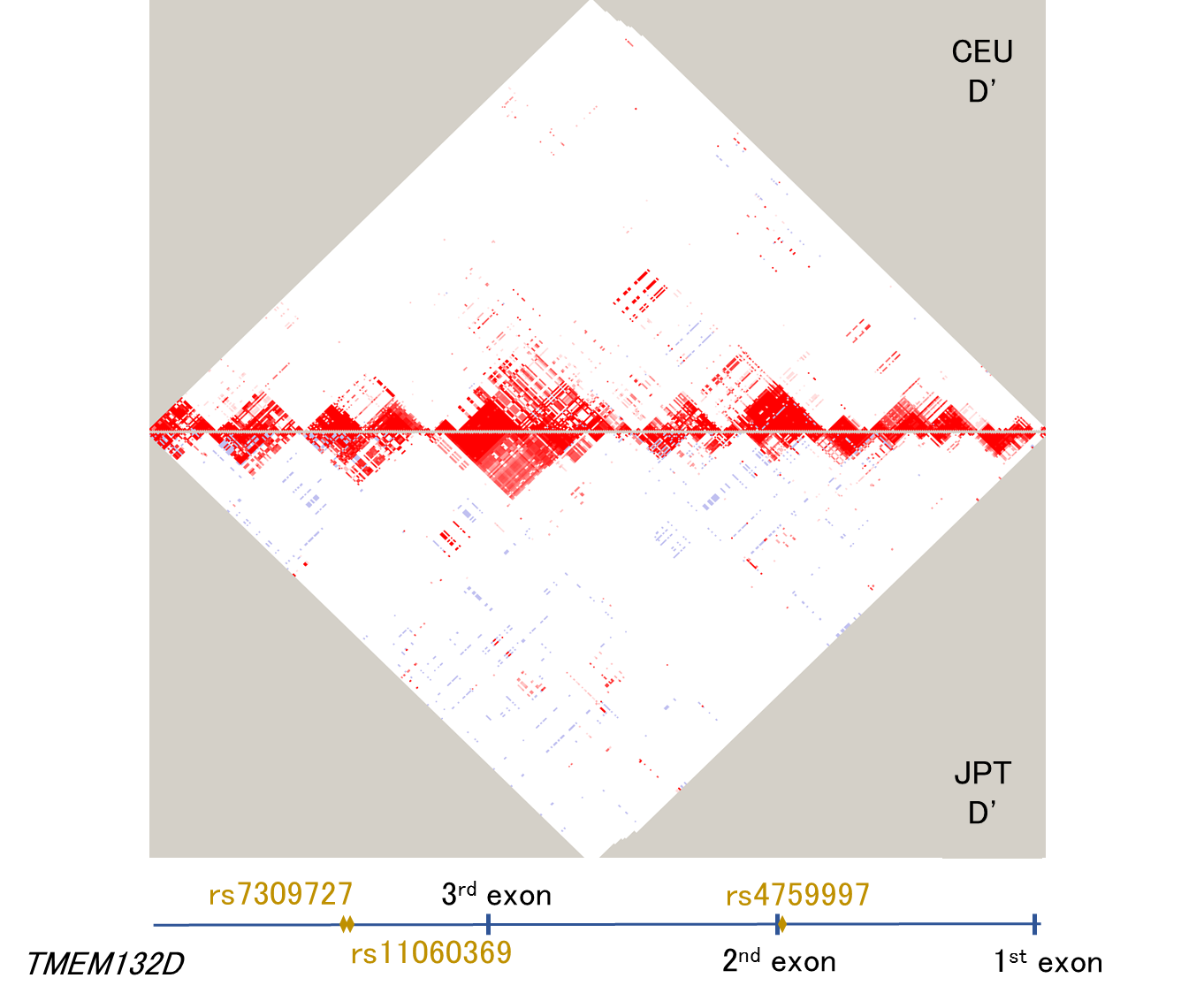


Supplementary Figure 3. Pairwise linkage disequilibrium (D’) diagram for the *TMEM132D* intron 1-3 region (chr12:129,864,100 - 130,400,300 (GRCh37/hg19)) of Japanese and European populations.

The linkage disequilibrium (LD) blocks with SNPs (MAF ≧ 20% and HWE P ≧ 0.05) were shown using Haploview. The SNP genotype data of HapMap database (HapMap Data Rel 27 Phase II+III, Feb09) were used for the analysis.
